# Preserved force control by the digits via minimal sparing of cortico‐spinal connectivity after stroke

**DOI:** 10.1113/EP092134

**Published:** 2024-12-14

**Authors:** Michael A. Urbin, Fang Liu, Chan Hong Moon

**Affiliations:** ^1^ Human Engineering Research Laboratories, VA RR&D Center of Excellence VA Pittsburgh Healthcare System Pittsburgh Pennsylvania USA; ^2^ Department of Physical Medicine & Rehabilitation University of Pittsburgh Pittsburgh Pennsylvania USA; ^3^ Department of Radiology University of Pittsburgh Pittsburgh Pennsylvania USA

**Keywords:** cerebrovascular accident, diffusion MRI, electrophysiology, motor control, noninvasive stimulation, stroke

## Abstract

The ability to regulate finger forces is critical for manipulating objects during everyday tasks but is impaired after damage to white matter tracts that transmit motor commands into the spinal cord. This study examines cortico‐spinal connectivity required for force control by the digits after neurological injury. We report on a unique case of a stroke survivor who retained the ability to control finger forces at a level comparable to neurologically intact adults despite extensive loss of white matter volume and severely compromised transmission from cortical motor areas onto the final common pathway. Using a combination of imaging methods and noninvasive stimulation techniques, we illustrate the structure and function of a slow‐conducting, cortico‐spinal pathway minimally spared by stroke that underlies this stroke survivor's ability to transition and stabilize finger forces of the paretic hand during precision grip. We interpret findings in the context of physiological mechanisms underlying distal limb control and current thinking on neural adaptation after brain injury due to stroke.

## Abstract (highlights)

- **What is the central question of this study?**
  What is the minimum neural substrate needed to regulate voluntary contractions of intrinsic hand muscles principally under monosynaptic control?
- **What is the main finding and its importance?**
  A cortico‐spinal system marginally spared by neurological injury can support force control by the digits that is within the range of performance by neurologically‐intact humans, indicating that minimal surviving neural substrate has the potential to support distal limb control.

## 1 INTRODUCTION

Neurological injury can induce structural and functional adaptation at multiple levels of the neuraxis (Urbin, 2025). Cellular through network‐wide pathophysiology has been studied extensively to better understand adaptation patterns that support  distal limb control after injury. Despite progress made to this point, there is still much to be learned about physiological mechanisms underlying motor control to inform therapeutic strategies that aim to reconnect the cortical origin of descending motor commands with spinal motor neuron pools innervating skeletal muscle.

Cases of neurological injury tend to be highly heterogeneous, which complicates efforts to identify and dissociate adaptation patterns that underlie the capacity to recover gross upper limb movements used to reach and grasp for objects and finger movements used to manipulate them. Tools available to study structure and function of the human nervous system are also not without limitation. These issues aside, a more basic consideration that can be overlooked relates to *how much* residual neural substrate is needed to support limb control after injury. Brain injury due to stroke, for example, can lead to varying degrees of loss in descending white matter tracts that are the structural medium by which supraspinal input is transmitted to influence the behaviour of spinal motor units, particularly for intrinsic hand muscles principally under monosynaptic control (Muir & Lemon, 1983).

Force stability is primarily a function of motor unit recruitment at low force levels (Duchateau et al., 2006). Spinal motor unit recruitment (Urbin et al., 2021) and the ability to grade and stabilize force by the digits (Lafe et al., 2023) are compromised by brain injury due to stroke. Stroke is a leading cause of disability (Feigin et al., 2021) with high rates of upper limb dysfunction (Lawrence et al., 2001), yet low rates of recovery (Jørgensen et al., 1999). The minimal amount of connectivity needed to preserve force control by the digits, however, remains poorly understood. Given the heterogeneity of this clinical population, clearly defining the minimum residual cortico‐spinal structure and function needed to support distal limb control has the potential to inform which pathophysiological profiles stand to gain from motor retraining at the chronic stage of stroke when disability tends to persist. The aim of this case report is to demonstrate that approximately normal force control by the digits remains possible when cortico‐spinal connectivity is minimally spared and to characterize different aspects of physiology underlying preservation of this ability.

## 2 CASE DESCRIPTION

A 49‐year‐old male who sustained a stroke at the age of 31 years presented to our research laboratory for enrollment in a study on longstanding hand impairment following stroke. The subject provided signed informed consent to undergo research procedures approved by the Institutional Review Board at VA Pittsburgh Healthcare System in accordance with guidelines established by the *Declaration of Helsinki*. Review of the medical record indicated that the subject was unable to speak, had a partial right visual field cut, and exhibited uniform weakness on the right side of the body upon admission to the emergency room. Muscle power was less than antigravity strength in the right arm and leg, but reflexes were symmetric. Loss of grey/white matter differentiation and sulcus effacement with a hypodensity in the third middle cerebral artery (MCA) territory was observed on computed topography. The subject was diagnosed with acute stroke due to left MCA occlusion and met criteria for intervention with intravenous tissue plasminogen activator, which was administered approximately 2.5 h after symptom onset. The occlusion did not resolve, and craniotomy was required to accommodate swelling.

Nearly two decades later when this stroke survivor presented to our laboratory, manual muscle testing showed a reduction in muscle power of the right arm, which the survivor identified as his dominant side prior to stroke onset. There was a slight increase in muscle tone with minimal resistance at the end of the range of motion in both elbow and wrist flexors/extensors, but the shoulder and elbow were fully extended as part of his preferred seated and standing posture. A video captured by the subject's family is included in Supporting information, showing him perform resisted movements resisted movements with the paretic arm shortly after the approximate date he underwent testing. He was able to ambulate without assistance, albeit with abnormal gait characteristics due to right‐sided paresis. The subject reported taking no anti‐spasticity medications in the years preceding enrollment in the study.

**FIGURE 1:**
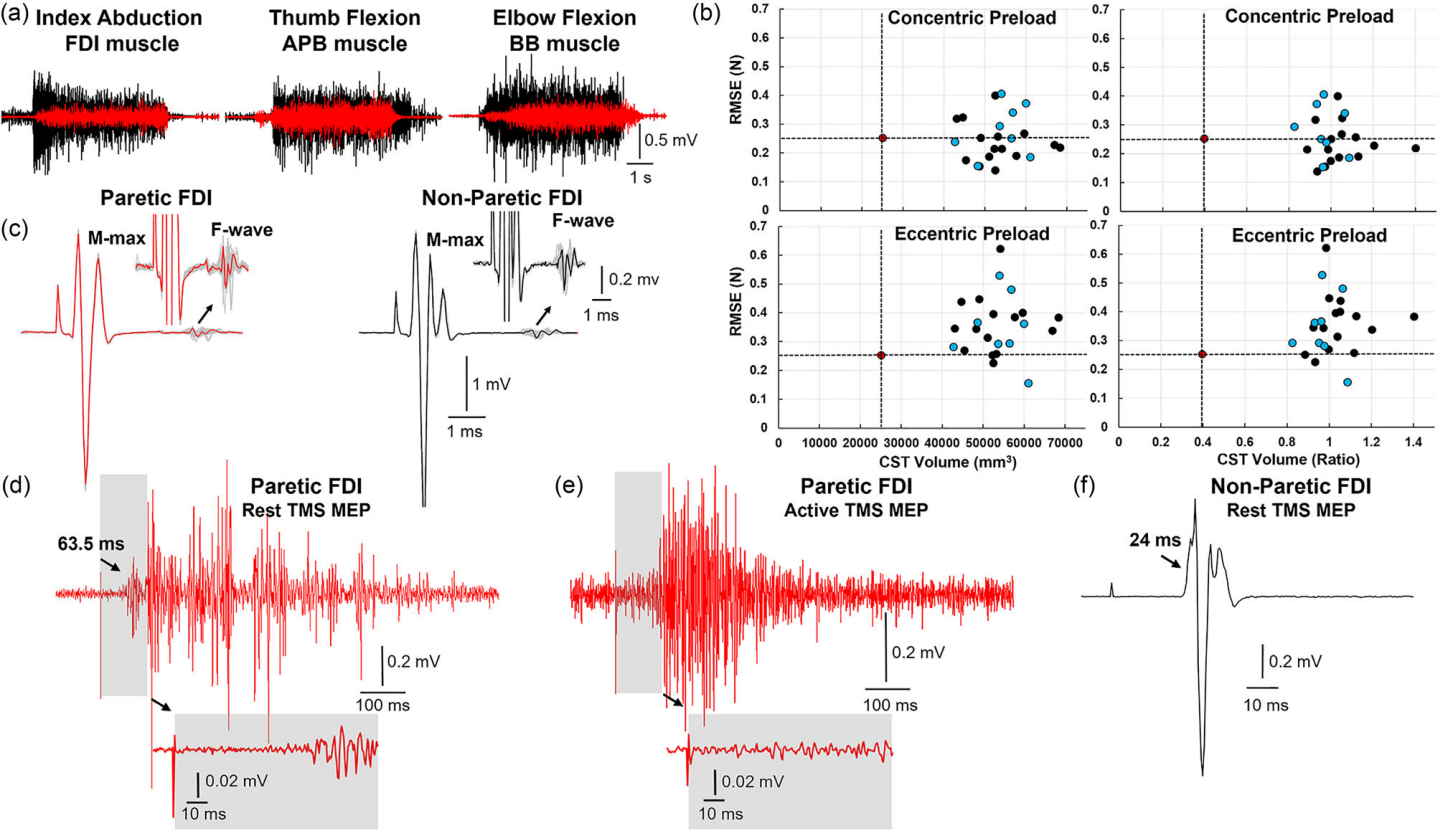
Electrophysiological recordings and white matter volume versus force control during precision grip. (a) EMG from non‐paretic (black trace) and paretic (red trace) muscles during maximal voluntary contractions. (b) Scatter plots depicting RMSE in Newtons versus white matter volume non‐normalized (left column, dominant side) and normalized between sides (right column, non‐dominant/dominant). RMSE is a measure of variability about the target force with lower values corresponding to greater stability after generating (top row) or releasing (bottom row) force. The stroke survivor is represented by the red data point intersected by the dashed lines, and controls are represented by black data points (blue data points for age‐matched controls). (c) M‐ and F‐wave recordings from the paretic (red) and non‐paretic (black) FDI muscle. (d,e) EMG recordings from the resting (d) or active (e) paretic FDI muscle showing the response elicited by single‐pulse TMS. The early portion of the response observed in the resting FDI is absent in the active FDI (gray shaded region). (f) EMG recordings from the resting non‐paretic FDI muscle showing the response elicited by single‐pulse TMS. EMG, electromyographic; FDI, first dorsal interosseous; RMSE, root mean square error; TMS, transcranial magnetic stimulation.

**FIGURE 2:**
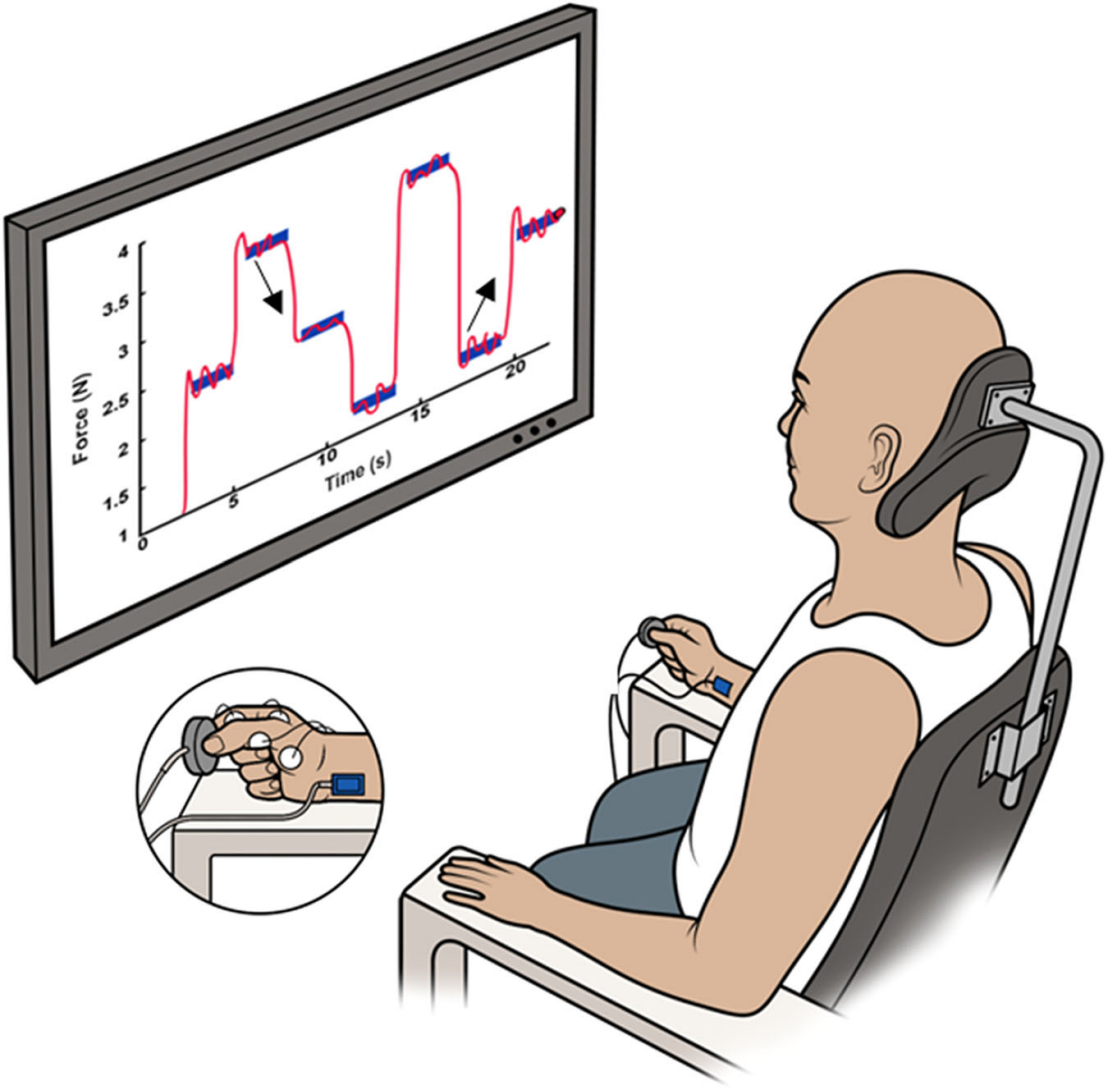
A visuomotor task was used to evaluate the stroke survivor's ability to control finger forces from concentrically and eccentrically preloaded muscle states. The arrow pointing up represents a concentric preload, and the arrow pointing down represents an eccentric preload.

Electromyographic (EMG) recordings showed clear voluntary muscle activation in the first dorsal interosseous (FDI), abductor pollicis brevis (APB) and biceps brachii (BB) muscles during maximal contractions into index finger abduction, thumb flexion and elbow flexion, respectively (Figure 1a). Both generating and releasing forces applied by the digits contribute to hand dexterity after stroke (Pennati et al., 2020; Plantin et al., 2022). This dynamic control of force is needed to manipulate objects held between tactile pads of the thumb and index finger during everyday tasks (e.g., buttoning a shirt, writing with a pen, or cutting with a knife). We tested the subject's capacity to control finger forces using a visuomotor task paradigm that approximates transitions in low‐level forces used during precision grip. The visuomotor task paradigm required the subject to shorten (i.e., concentric) or lengthen (i.e., eccentric) intrinsic hand muscles under tension prior to stabilizing force (Figure 2). In this way, we characterized control of finger movements during a functionally relevant task and constrained recruitment of muscles to those readily studied with noninvasive stimulation techniques. A cross‐sectional study of stroke survivors and controls using the visuomotor task has been reported previously (Lafe et al., 2023) and details on methodology are reported in Supporting information Section 1.

The level of force control exhibited by the subject from concentrically and eccentrically preloaded muscle states was in the 47th and 91st percentile, respectively, of a neurologically‐intact sample of adults (*n* = 23, 58.3 ± 10.7 years, 11 males, 22 right‐hand dominant) (Figure 1b). There was no correlation between age and concentric (*r *= −0.20) or eccentric (*r *= 0.25) preloads in our sample. However, the survivor was in the 50th and 88th percentile, respectively, after reducing the sample to controls who were within 5 years of his age or younger (*n* = 8, 45.8 ± 6.9 years, 6 males, all right‐hand dominant) (blue data points in Figure 1b).

Electrical pulses applied to the ulnar nerve elicited clear M‐ and F‐waves in the FDI muscle, indicating preserved orthodromic and antidromic propagation along peripheral axons (Figure 1c). Measures of F‐wave size and persistence are thought to reflect the intrinsic excitability of lower motor neuron pools (Thomas et al., 2017), but more recent work has demonstrated a contribution from interneuronal circuits to the F‐wave (Özyurt et al., 2024). Both maximal M‐wave peak‐to‐peak amplitude (paretic = 4.68 mV; non‐paretic = 5.22 mV) and average F‐wave peak‐to‐peak amplitude (paretic = 128 µV; non‐paretic = 94 µV) were comparable between sides. F‐wave persistence was identical (95%) in the paretic and non‐paretic FDI. Attempts to elicit H‐reflexes were unsuccessful despite the presence of spontaneous motor unit discharges in some instances throughout testing procedures, limiting a determination of spinal excitability.

Given evidence of preserved finger force control and symmetry in M‐wave size between sides, single‐pulse transcranial magnetic stimulation (TMS) was used to elicit currents in the canonical direction (i.e., posterior‐to‐anterior) within M1 to obtain recordings of motor‐evoked potentials (MEPs) from hand muscles. The subject was seated in an upright position with the forearm resting in a prone position on an arm tray. Stereotaxic neuronavigation was used to locate and register the optimal stimulation site contralateral to the paretic hand. TMS pulses (*n* = 7/10) applied over a relatively focal scalp site with near‐maximal stimulator output (90%) elicited abnormal, oscillatory movements of the wrist that lasted a few seconds before subsiding. Follow‐up attempts to manually induce clonus were unsuccessful. After verifying that the subject was alert, comfortable and willing to continue, additional TMS pulses were applied and most (*n* = 8/10) elicited the same response. EMG recordings revealed distinct bursts of activity in the FDI muscle that began approximately 63.5 ms after stimulation onset (Figure 1d). Aside from an unusually long latency, the initial waveform also did not appear consistent with a typical, triphasic MEP and was somewhat distinct from subsequent bursts of activity.

Single‐pulse TMS in a pre‐contracted muscle suppresses voluntary muscle activity, which is mediated by γ‐aminobutyric acid (GABA_B_) receptors in M1 (Werhahn et al., 1999). The presence of a silent period, therefore, was intended to confirm that the abnormal response occurring 63.5 ms after stimulation onset was transynaptically mediated in M1. The stroke survivor was instructed to press tips of the index finger and thumb together as single‐pulse TMS was applied to the same scalp site and with the same stimulator output used while the hand muscles were relaxed. Neither the initial waveform nor a silent period was observed, but there was a facilitation occurring ∼100 ms after stimulation onset, or ∼30–40 ms later than the initial burst of activity observed in the resting FDI muscle (Figure 1e). The optimal stimulation site contralateral to the non‐paretic hand was located quickly through use of low stimulator outputs (resting motor threshold = 37%), and M1 waveforms in the FDI muscle appeared normal in size, shape and latency (Figure 1f). On the basis of these initial findings, the origin and neural element(s) mediating the abnormal response by single‐pulse TMS contralateral to the paretic hand could not be determined.

**FIGURE 3:**
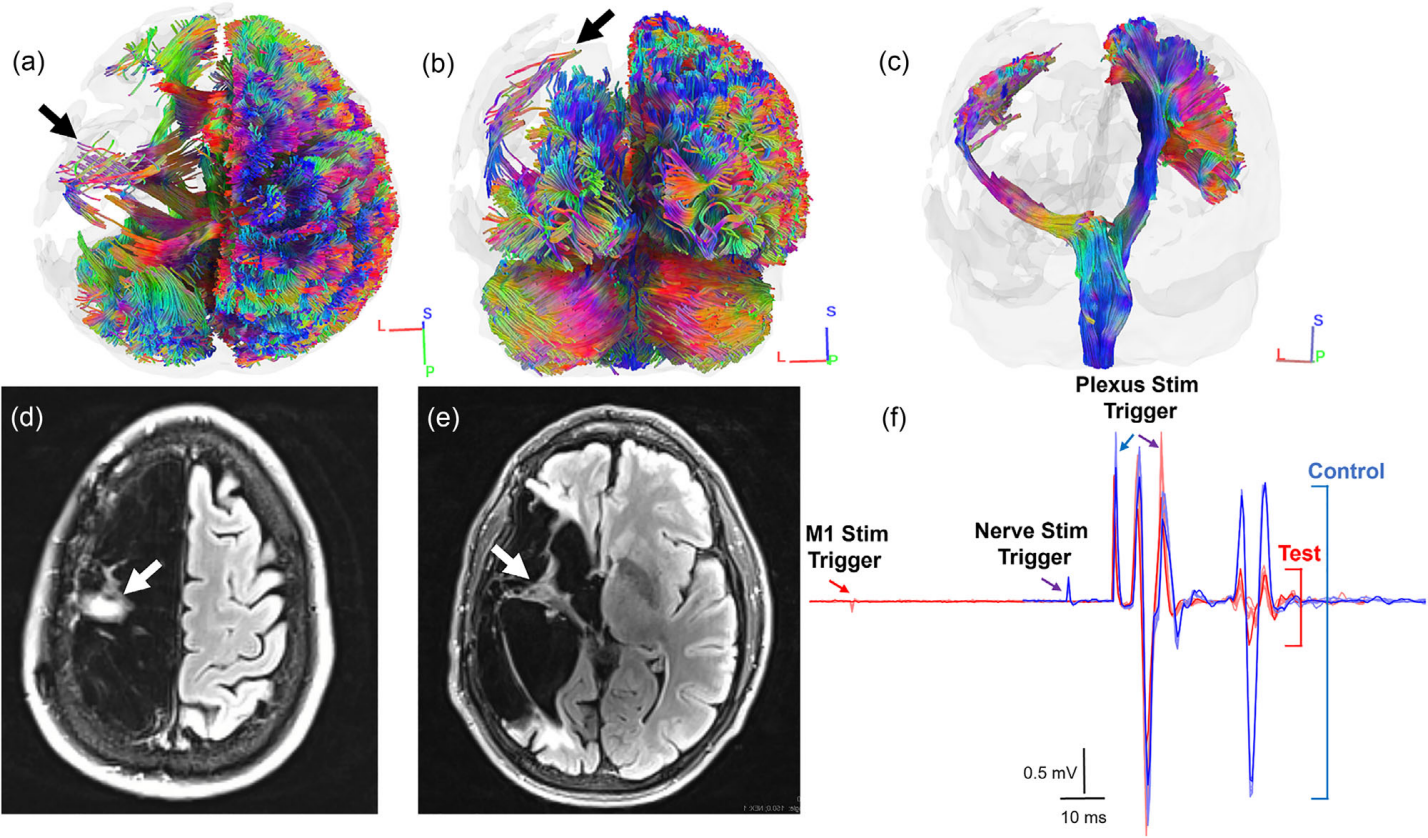
Whole brain tractography versus FLAIR imaging and cortico‐spinal recruitment of the spinal motor neuron pool. (a,b) Axial (a) and coronal (b) views of whole‐brain tractography. (c) Reconstructed residual (left) and intact (right) tracts. (d,e) Axial view of FLAIR images at more rostral (d) and caudal (e) slices. Note the correspondence between white matter in the stroke‐affected hemisphere observed on whole‐brain tractography and FLAIR imaging (arrows). (f) The ratio between test (red) and control (blue) curves is taken to reflect the overall proportion of the lower motor neuron pool brought to threshold by upper motor neurons. Multiple test and control curves are overlaid. Test curve amplitude is considerably reduced relative to control curve amplitude, but the presence of the test curve indicates that connectivity between upper and lower motor neurons is preserved. FLAIR, fluid attenuated inversion recovery.

The stroke survivor was brought in for MRI, and diffusion spectrum imaging data were analysed with an approach that has been used previously to reconstruct the residual descending corticofugal projection from M1 in individuals with longstanding stroke (Lafe et al., 2023). Details on methodology are reported in Supporting information, Section 2. Whole brain tractography revealed a significant loss of white matter volume in the stroke‐affected cerebral hemisphere and sparse descending fibres from the approximate location of M1 (Figure 3a,b). The orientation of these fibres was more lateral and their trajectory through the internal capsule was appreciably more oblique relative to fibres in the opposite cerebral hemisphere (Figure 3c). Noting the unusual appearance of the tract, we searched the medical record and found a fluid attenuated inversion recovery (FLAIR) MRI obtained about 1 year after stroke onset. This image showed white matter in the same approximate location, confirming results of our tractography analyses (Figure 3d,e). There was 76.8% white matter volume loss within the damaged portion of the residual tract, which was the third greatest loss observed in a sample of stroke survivors with longstanding hand impairment (*n* = 25) who were studied with the same procedure used here. There also was a notable asymmetry in total white matter volume of the entire corticofugal projection (39.7%), which was considerably less than in controls (99.1 ± 10.3%, non‐dominant relative to dominant tract). After parsing portions of the corticofugal projection into precentral gyrus and remaining subcortical white matter, a pronounced asymmetry in cortical white matter volume (37.7%) was observed relative to controls (99.9 ± 12.9%). Subcortical white matter volume was also considerably reduced (40.2%) relative to controls (99 ± 10.7%), but the relative proportion of subcortical volume comprising the entire projection was approximately symmetrical between sides (101%) and comparable to controls (100 ± 1.7%).

**FIGURE 4:**
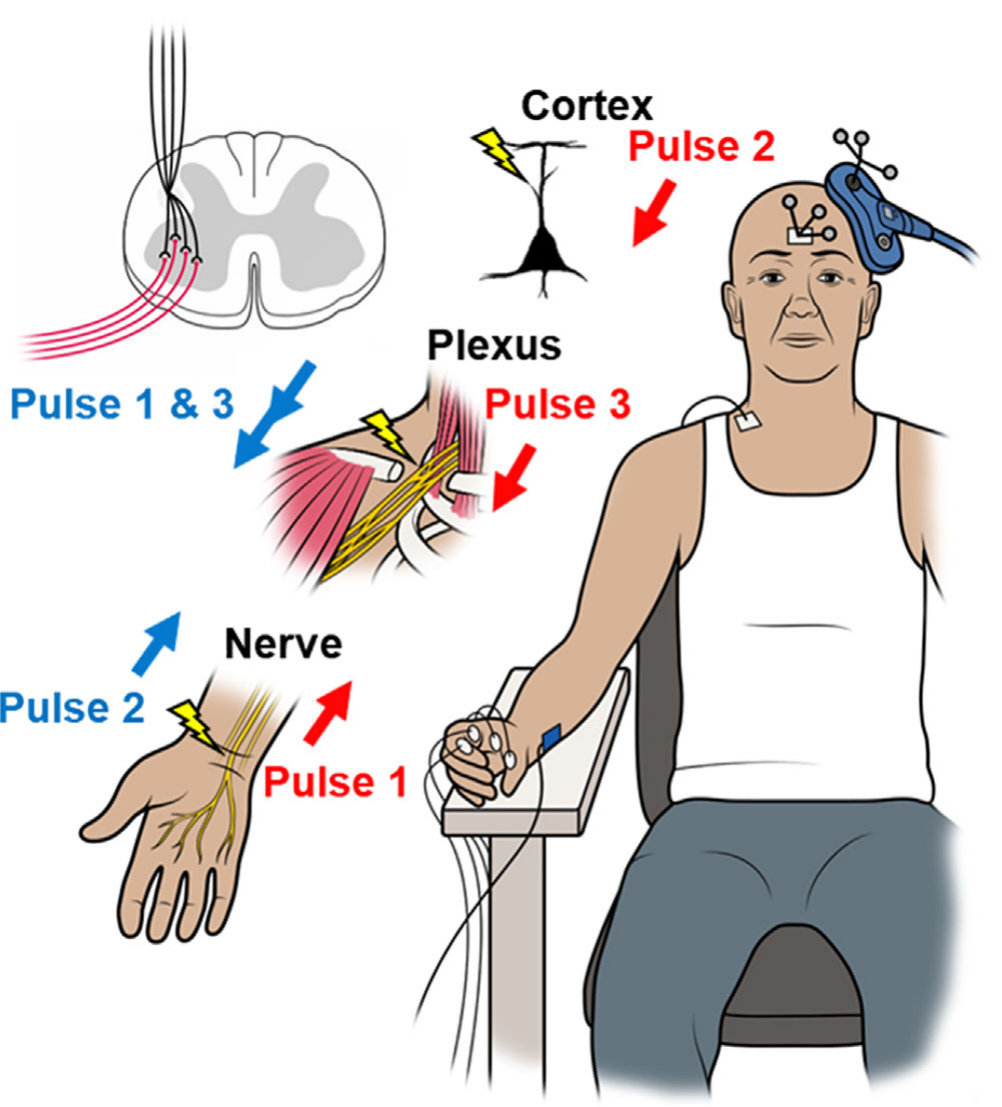
Triple‐pulse stimulation was used to collide volleys along peripheral axons and quantify cortico‐spinal recruitment of spinal motor neurons. Two separate sets of three pulses (blue, control curve; red, test curve) are triggered to activate neural elements at predetermined latencies in the order shown. The size of the electrophysiological response elicited by the third pulse in the test curve is expressed as a percentage of the same in the control curve and used to quantify spinal motor neuron recruitment.

Aside from clear structural abnormalities of the residual tract, both MEP latency (paretic FDI = 63.5 ms; non‐paretic = 24.5 ms) and central conduction time (paretic = 48 ms; non‐paretic = 6.5 ms) were highly asymmetric. The level of force control exhibited by this stroke survivor relative to controls was deemed rather remarkable considering the minimal amount of surviving white matter and asymmetries in conduction. Stated another way, the abnormal appearance and reduced density of descending outflow from M1, as well as the extremely long latency and aberrant nature of the response to single‐pulse TMS, did not seem to comport with a nervous system capable of supporting the level of force control observed in this case. To determine how much of the lower motor neuron pool could be accessed via this slow‐conducting pathway, the survivor was brought back for additional testing. A triple stimulation technique was administered to approximate the proportion of the spinal motor neuron pool brought to threshold by cortical motor neurons (Figure 4). This technique has been used with stroke survivors previously (Urbin et al., 2021), and details on methodology are reported in Supporting information, Section 3. Recruitment of lower motor neurons on the paretic side (Figure 3f, 17.9%) was evident but considerably reduced relative to the non‐paretic side (79.6%).

## 3 DISCUSSION

The stroke survivor documented in this case report was capable of controlling finger forces during precision grip well within the range of neurologically‐intact individuals. This ability was unexpected given the extent of damage and highly abnormal appearance of the residual corticofugal projection from M1. Connectivity with the spinal cord was spared, yet highly compromised via this slow‐conducting pathway, as single‐pulse TMS activated surviving cortical motor neurons that retained access to a small portion of the spinal motor neuron pool. The strength of supraspinal input was insufficient to inhibit reflexive activity in spinal circuits, leading to repetitive discharges of lower motor neurons that manifested as a clonus‐like behaviour of the upper limb. EMG recordings from intrinsic hand muscles showed an activation pattern elicited by stimulation that was comprised of an initial, lower‐amplitude burst followed by multiple, higher‐amplitude bursts. Oscillatory spikes in the EMG signal following the initial response appear consistent with a mechanoreceptor‐mediated response involving a transcortical pathway through M1 (Pruszynski, 2014). Specifically, initial excitation of muscle fibres may have generated somatosensory receptor potentials in the periphery, eliciting activity in reflex circuits that reverberated across the neuraxis. Reduced sensory gating at one or more levels of the neuraxis (Seki & Fetz, 2012) due to the severity of damage may have contributed to the inability to suppress activity in local reflex circuits. Lack of the abnormal motor response or a silent period but gradual facilitation of activity when stimulation pulses were applied with the hand muscles in a pre‐contracted state also appears consistent with this interpretation. Taken together, the data suggest a reduced ability of supraspinal input to excite the spinal motor neuron pool and inhibit local spinal circuits. Despite compromised transmission along a minimally spared corticofugal projection from M1, this stroke survivor retained the ability to control muscle contractions underlying finger movements in a way that was normal.

Recovering control of the upper limb following stroke depends on integrity of the damaged, contralateral corticospinal tract (Byblow et al., 2015), but there is evidence supporting a contribution of pathways descending ipsilateral to the paretic limb. Given the limited proportion of direct, ipsilateral corticospinal projections to spinal motor neurons in both human (Palmer et al., 1992) and non‐human (Rosenzweig et al., 2009) primates, an indirect cortico‐reticulo‐spinal pathway generally has been considered a more plausible mediator of descending motor commands (Bradnam et al., 2013). The reticulospinal tract is upregulated in the macaque 6 months after pyramidal tract lesions (Zaaimi et al., 2012), and increased ipsilateral connectivity to paretic arm muscles is observed in humans with longstanding stroke (Choudhury et al., 2019; Taga et al., 2021). The rubrospinal tract is another phylogenetically older descending pathway originating in the midbrain red nucleus. Studies have shown potential for this extrapyramidal tract to provide a compensatory mechanism for distal limb control after corticospinal lesions in the macaque (Belhaj‐Saïf & Cheney, 2000), and there is also evidence that red nucleus microstructure is correlated with distal limb function in human stroke survivors (Rüber et al., 2012; Takenobu et al., 2014). Propriospinal relays in the macaque cervical cord contribute to force control during precision grip (Takei & Seki, 2013), providing another possible alternative pathway for transmitting motor commands to spinal motor neurons after stroke (Tohyama et al., 2017).

Although the descending pathway(s) mediating communication between cortical and spinal motor neurons after stroke is not fully resolved, unique adaptations have been observed *postmortem* in humans who sustained neurological injury early in life. One example is a compensating hypertrophy in the medullary pyramid contralateral to cerebral injury (Scales & Collins, 1972). Our diffusion MRI approach is a much less direct means to study the pyramids relative to techniques used as part of *post* *mortem* investigations. However, the tracts that we reconstructed are consistent with structural images from the medical record that were obtained much closer to the time of stroke onset and appear to show an increase in white matter volume at the caudal end of the corticofugal projection contralateral to the injured side (Figure 3c). The asymmetry in white matter volume clearly extends below the level of cortex, with more caudal portions of the tract appearing more symmetrical. Whether this observation is consistent with compensating hypertrophy in an individual who sustained injury at a fairly young age, yet likely after full neural maturation is less clear. Regardless, the principal outflow of descending motor commands was severely diminished but still retained the ability to transmit neural impulses along a minimally spared corticofugal projection onto the final common pathway to control the digits. Spinal motor neuron pools remain morphometrically unaltered after brain injury due to stroke (Qiu et al., 1991; Terao et al., 1997), which appears consistent with electrophysiological findings (i.e., symmetry of M‐wave size) in this case. The reticulospinal and rubrospinal tracts originate in the brainstem, but the former is thought to be involved in more gross aspects of limb control (Glover & Baker, 2022) and evidence that the latter projects below the level of the third cervical segment in humans is lacking (Nathan & Smith, 1982). Nevertheless, the relative distribution of white matter volume observed in this case is interesting given the survivor's capacity to control contractions of intrinsic hand muscles that are thought to be under monosynaptic control (Lemon, 2019).

Tools used to probe pathophysiology here demonstrate that both cortico‐spinal structure and function were significantly compromised by a single cerebrovascular accident that occurred many years earlier in this stroke survivor. His ability to control finger movements despite extensive neurological damage seems to instantiate the nervous system's adaptive capacity. Left with only a fraction of the tract mediating voluntary motor control, he was able to walk unassisted and regulate forces by the digits similar to individuals without neurological injury. This single case study has inherent limitations, including the lack of longitudinal data and the inability to generalize findings to the broader population. Longitudinal studies involving larger cohorts are needed to validate these findings and to identify specific mechanisms underlying motor recovery after severe neurological injury.

## AUTHOR CONTRIBUTIONS

Michael A. Urbin conception or design of the work. Michael A. Urbin, Fang Liu and Chan H. Moon acquisition, analysis, or interpretation of data for the work, and drafting of the work or revising it critically for important intellectual content. All authors have approved the final version of the manuscript and agree to be accountable for all aspects of the work in ensuring that questions related to the accuracy or integrity of any part of the work are appropriately investigated and resolved. All persons designated as authors qualify for authorship, and all those who qualify for authorship are listed.

## CONFLICT OF INTEREST

None declared.

## Supporting information



Supporting video

Supporting information

## Data Availability

The data that support the findings of this study are available from the corresponding author upon reasonable request.
