# Supplementary figures and images for: Preserved force control by the digits via minimal sparing of cortico‐spinal connectivity after stroke
Source: Exp Physiol. 2024 Dec 14;110(3):363–9. doi: 10.1113/EP092134 (PMC11868025; doi:10.1113/EP092134)

## Slide 1
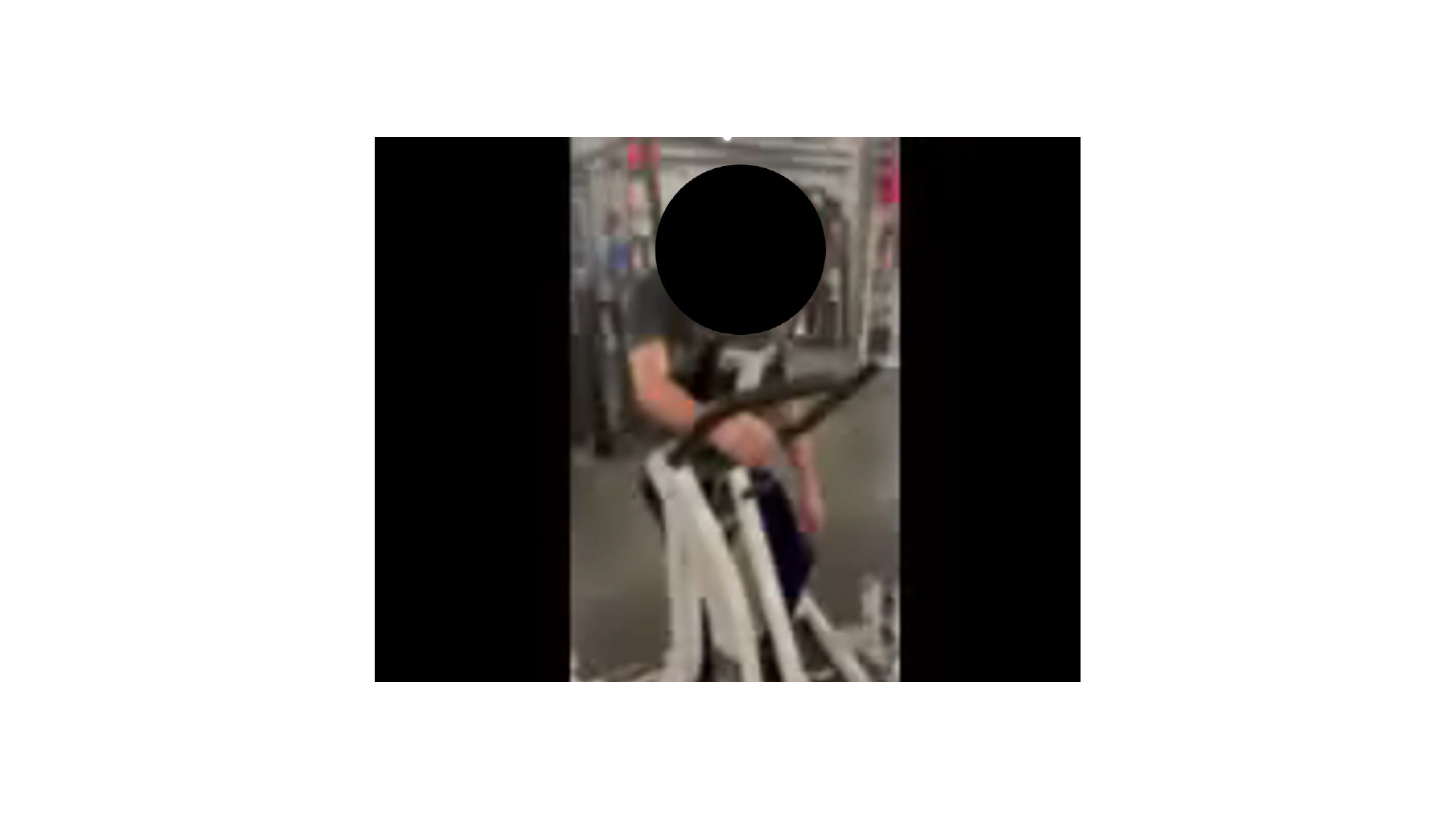

Supplement: Supplementary file 1 — Supporting video [file EPH-110-363-s002.pptx]
