# Supplementary material for: Preserved force control by the digits via minimal sparing of cortico‐spinal connectivity after stroke
Source: Exp Physiol. 2024 Dec 14;110(3):363–9. doi: 10.1113/EP092134 (PMC11868025; doi:10.1113/EP092134)
Supplement: Supplementary file 2 — Supporting information [file EPH-110-363-s001.docx]

**Supplementary Information**

Preserved force control by the digits via minimal sparing of cortico-spinal connectivity after stroke

**Rationale**

Skillful manipulation of objects during everyday tasks requires control of the forces exerted on them by the digits. A visuomotor task paradigm described in **Section 1** and illustrated in **Figure 2** was used to study force control by the digits during precision grip. Intrinsic hand muscles are involved in precision grip, and electrophysiological responses elicited by noninvasive stimulation in these muscles can be used to study the contribution of different neural elements within descending pathways that transmit motor commands from cortical motor areas into the spinal cord. The latency and size of motor-evoked potentials (MEPs) elicited by single-pulse transcranial magnetic stimulation (TMS) generally occur within a fixed range (Vallence *et al.*, 2023), and the time required for descending volleys to propagate from cortex onto spinal motor neuron pools also does not vary considerably (Udupa & Chen, 2013). Characteristics of F-waves elicited by supramaximal stimulation of a peripheral nerve are used to make inferences about the intrinsic excitability of spinal motor neurons (Christiansen *et al.*, 2018). Although F-wave characteristics were found to be symmetric between sides in the stroke survivor studied here, MEP latency and central conduction time were far outside of the ranges observed in severe cases, creating uncertainty about the pathway(s) mediating the rhythmic bursts of muscle activity elicited by single-pulse TMS.

A methodological consideration in neuroimaging analyses of longstanding stroke is how to handle cases where large portions of brain parenchyma no longer exist due to pathophysiological processes that ensue after neurological injury, which can lead to significant atrophy in white matter. The method described in **Section 2** was used to capture all remaining fibers descending from primary motor cortex (M1) using diffusion MRI. Diffusion spectrum imaging (DSI) enables more robust reconstruction of fiber tracts relative to diffusion imaging because it captures a more complete diffusion probability distribution within each voxel, thus, resolving multiple diffusion directions per voxel (Kamagata *et al.*, 2024). Tractography revealed a sparse residual corticofugal projection from M1 in the stroke survivor studied here. Aside from the sparsity of fibers, the residual tract’s trajectory was unusual which also did not resolve uncertainty about the motor response elicited by single-pulse TMS. Radiological evidence from the medical record was sought for verification, and an image obtained using an inversion recovery sequence showed a residual tract that closely approximated the one reconstructed by tractography analyses, confirming that the rhythmic bursts of muscle activity elicited by single-pulse TMS were mediated along this pathway.

Although MEP size is in some way related to hand function after stroke (Buetefisch *et al.*, 2018), the highly irregular bursts of muscle activity that occurred over several hundred milliseconds in the stroke survivor studied here did not allow for a straightforward comparison. Muscle weakness after stroke is largely a result of upper motor neurons in cortex losing access to lower motor neurons in the spinal cord (Sathian *et al.*, 2011). A collision technique described in **Section 3** and illustrated in **Figure 4** quantifies how much of the spinal motor neuron pool innervating a muscle is recruited by cortical motor neurons (Magistris *et al.*, 1998). Results obtained with this technique confirmed that the residual M1 could still activate spinal motor neuron pools innervating paretic intrinsic hand muscles, though recruitment was reduced considerably relative to the intact side.

**Section 1: Visuomotor Task Paradigm: Force Control by the Digits**

*Setup*

The visuomotor task paradigm was created in MATLAB version 2021a (The MathWorks, Inc). Subjects sat in a chair approximately 1.16 m in front of a computer monitor (36.5 cm height × 61.4 cm width, 3840 horizontal pixel × 2160 vertical pixel resolution, 144 Hz refresh rate), resulting in a ~17.9° vertical × ~29.7° viewing angle. Force signals were sampled at 200 Hz using a 6-axis force sensor (Mini40, ATI Industrial Automation). The pixel-to-Newton ratio was set at 64 pixels/N such that the visual indicator for force would move 64 pixels higher on the vertical axis of the monitor for every Newton of force applied along the z-axis of the sensor. The forearm was maintained in a neutral position on an arm tray, and the sensor was held between the index finger and thumb with remaining digits fully flexed. Colored tape was placed on the sensor to indicate which side to place the index finger and thumb. Subjects gripped the sensor and maintained forearm position in the same way throughout testing.

A calibration procedure was administered at the start of testing as a warm up and

to familiarize subjects with holding the sensor and stabilizing force. Measurements obtained from the calibration procedure were used to establish target templates. The first step of the calibration procedure defined the noise floor of the sensor by sampling data while it was positioned on a stable surface. Next, subjects grasped the sensor and were verbally exhorted by the experimenter to produce a true maximum voluntary contraction (MVC). Precision grip MVC was recorded over 5 s and taken as the mean value calculated from 2 s centered around the peak force. Subjects then performed a single attempt at matching forces corresponding to 10%, 20%, 30%, 40%, and 50% of their MVC over 5 s. The display window showed a horizontal line at the force level to be matched while subjects attempted to stabilize force represented by the height of a second, horizontal line that corresponded to the force applied to the sensor. After a brief rest period, the calibration procedure was repeated a second time. Measurements obtained from this second calibration procedure were used to establish force values (vertical positions) and accuracy tolerances (vertical widths) for the set of targets displayed during testing.

The visuomotor task required subjects to increase or decrease the amount of force applied on the sensor to control a circular red cursor (10-pixel diameter) in an upward or downward direction, respectively, as it moved across the display window at a constant rate. The objective was to control dynamic and static contractions of intrinsic hand muscles to align the path of the cursor through the center of the target forces represented by blue rectangles. Target force values were set at 7%, 14%, and 21% of MVC to approximate the low-level forces used during precision grip and also to ensure that the maximum difference between lower and upper force targets for subjects with large MVCs did not exceed viewing limits on the display window. Vertical width of the target at each respective target force was set by regressing variability in the force signal (i.e., 1 standard deviation) from the final 2 s of recordings (i.e., where the error between the mean force and target force was minimized) onto each of the 5 force levels matched, with the intercept constrained to 0. Vertical width of each target, therefore, was constrained to the variability exhibited by each subject, requiring skilled control of finger movements to hold the cursor within target boundaries after generating or releasing forces applied on the sensor to transition from the previous target. This setup permitted force control to be characterized while intrinsic hand muscles were preloaded in either a concentric or eccentric state of contraction.

*Force Data Acquisition*

Subjects performed a 3 sets of 6 trials. A total of 7 target forces were presented simultaneously at the start of each trial. The red circle moved across the display window at a constant rate, resulting in a 22-s data sweep. The first target force was positioned 3 s into the sweep, allowing the subject adequate time to ramp force to its height. The interval between each set of adjacent targets (0.833 s) was a function of trial length (22 s), horizontal target length (2 s), and time of the initial target (3 s). This configuration was selected to approximate natural, dexterous actions that require force stability between transitory states while allowing a sufficient number of data points during each target crossing to quantify stability. The order of target force levels was pseudorandomized to ensure that (a) each force level was preceded by a different force level, resulting in 6 possible force level-to-force level transitions with each transition occurring only once within a trial; (b) each trial began and ended at the same force level to result in 3 target crossings at a single force level and 2 target crossings at each of the other force levels; and (c) the initial force level changed systematically such that each force level was presented as the initial target only once in a series of 3 trials.

*Force Data Processing*

Force signals were analyzed using MATLAB version 2021a. Signals from each target crossing (2-s time series, 400 samples) were detrended and filtered using a fourth-order Butterworth filter with a 12 Hz cutoff. Force stability was quantified using root-mean-square error (RMSE) to characterize variability around the center of the target force where n is the total number of force samples:

[Σ(Τ-*f*_i_)^2^/n – 1]^1/2^

RMSE, therefore, effectively measures variability about a straight line. Crossings at the 14% MVC target force were extracted and grouped according to force preload. In this precision grip task, intrinsic hand muscles are eccentrically loaded when transitioning from the 21% MVC target force to the 14% target force in order to maintain the sensor between the index finger and thumb. Eccentric preload, therefore, was defined as crossings at the 14% MVC target force preceded by crossings at the 21% MVC target force. Concentric preload was defined as crossings at the 14% MVC target force preceded by crossings at the 7% MVC target force. Given the previously described randomization scheme built into the visuomotor task paradigm, a total of 18 trials were extracted for each preload.

**Section 2: Tractography Analyses: Residual Corticofugal Projection from M1**

*Image Acquisition*

Subjects underwent MRI using a Prisma 3T scanner (Siemens, Erlangen, Germany) with a 64-channel head/neck receiver radio-frequency (RF) coil. Cushions were placed on either side of the face within the coil cage to restrict movement and to ensure bicommissural alignment. Structural MRI was acquired using T1 magnetization-prepared rapid acquisition with gradient-echo (MPRAGE) and T2 sampling perfection with application-optimized contrasts using a different flip angle evolutions (SPACE) sequence with the following parameters: repletion time (TR)/inversion time (TI)/echo time (TE) = 1900/900/1.67 ms, voxel resolution = 1.3-mm isotropic, acceleration factor = 2, acquisition time = 3 min 32 s; and TR/TE = 3200/412 ms, voxel resolution = 1-mm isotropic, acceleration factor = 2, acquisition time = 3 min 49 s, respectively). DSI data were acquired via a single-shot, twice-refocused, two-dimensional, multi-slice spin echo planar imaging (EPI) sequence (TR/TE = 2480/99.2 ms, voxel resolution = 2-mm isotropic, multi-band factor = 4, partial Fourier factor in phase encoding (PE) = 6/8, 258 diffusion directions with *b* values of 4000 s/mm^2^ and one with a *b*-value of 0 in the anterior-to-posterior direction; total acquisition time = 11 min). A separate sequence with a *b*-value of 0 was acquired (posterior-to-anterior PE direction, acquisition time = 19 s) to correct for spatial distortion in the diffusion weighted MR image

*DSI Data Processing*

DSI data preprocessing was completed using FSL (https://www.fmrib.ox.ac.uk/fsl) and included motion correction, eddy correction, as well as co-registration between T1, T2, MNI152 standard brain images with the three-dimensional DSI image. DSI studio software (https://dsi-studio.labsolver.org) was used to visualize fibers of the intact and residual tract in stroke survivors and both tracts in control subjects. A deterministic fiber tracking algorithm (angular threshold = 60◦, step size = 1 mm) was used to reconstruct corticofugal projections from M1 in both hemispheres to the caudal end of the image volume at the level of the brainstem based on subject-specific tract trajectories (Yeh *et al.*, 2013).

Although recent imaging evidence from humans indicates that corticospinal fibers arise from 9 different cortical areas that include ~50% from M1 and ~27% from premotor cortex (Usuda *et al.*, 2022), we aimed to capture fibers descending from M1 that mediate transmission of motor commands for distal limb control. Therefore, fibers were reconstructed using the co-registered precentral gyrus template from the Harvard-Oxford Cortical Structural Atlas and the Corticospinal Tract template from HCP842 Tractography Atlas as separate regions of interest. Fibers generated by seeding in both regions of interest were segmented separately by two independent raters who sought to retain only those fibers originating within M1 and descending into the internal capsule. Tracts segmented by both raters were merged prior to quantifying white matter volume in each cerebral hemisphere. Intraclass correlation coefficient estimates (ICC) and 95% confidence intervals (CI) were calculated based on absolute-agreement, two-way mixed-effects models using SPSS version 23 (SPSS Inc., Chicago). There was a high degree of agreement between raters on fiber number in stroke survivors [residual tract: (ICC = 0.960, 95% CI = 0.866 - 0.985); intact tract: (ICC = 0.981, 95% CI = 0.956 - 0.992)] and controls [dominant tract: (ICC = 0.930, 95% CI = 0.421 - 0.981); nondominant tract (ICC = 0.747, 95% CI = -0.090 - 0.922)].

We calculated the ratio of total white matter volume between the residual and intact tracts (stroke subjects) and dominant and non-dominant (control subjects) tracts, as well as the ratio of white matter volume contained within each precentral gyrus. Subcortical white matter volume was computed by subtracting the volume in precentral gyri from the total volume of the corresponding tract. Values were used to calculate the ratio of subcortical white matter volume. In stroke subjects, white matter volume loss was calculated within the damaged portion of the residual tract and expressed as a percentage of the volume in a mirror region within the intact tract.

**Section 3: Triple Stimulation: Lower Motor Neuron Recruitment**

*Setup*

Electromyography was recorded from the first dorsal interosseous (FDI) muscle through surface electrodes (Ag-AgCl, 10 mm diameter) secured to the skin in a muscle belly-tendon montage. The FDI muscle was selected because it is involved in precision grip (Johnston *et al.*, 2009; Larsen *et al.*, 2016), generating a flexion force about the metacarpophalangeal joint (An *et al.*, 1983). The skin was prepared with an abrasive cream and cleansed with alcohol prior to electrode placement. Signals were amplified, band-pass filtered (200–2000 Hz), and sampled at 2 kHz (Power1401, Signal, Cambridge Electronic Design Ltd., Cambridge, UK). A 200 Hz high-pass filter was used to aid in more accurately determining response latencies for colliding orthodromic and antidromic volleys along peripheral axons (Khan *et al.*, 2012). The subject sat in the same experimental chair used for testing on the visuomotor task.

*Transcranial Magnetic Stimulation*

Single monophasic, magnetic pulses intended to elicit posterior-to-anterior currents in M1 were applied to the scalp by a Magstim 200^2^ stimulator (The Magstim Company Ltd., Whitland, UK) through a figure-of-eight coil (70-mm loop diameter, D70). Currents elicited by single-pulse TMS in this direction transynaptically activate pyramidal cells in layer V of M1, producing volleys that descend along the corticospinal tract. Repeated discharge of lower motor neurons in response to the summation of descending volleys depolarizes the neuromuscular junction and is recorded as a MEP from the muscle. The 10–20 system was used to establish scalp locations corresponding to the vertex (Cz) and 7 cm lateral of Cz in line with the tragus of the ear contralateral to the target muscle. The optimal scalp site was determined by administering TMS pulses 7 cm lateral of Cz and moving the coil in ~1-cm increments in anterior–posterior/medial-lateral directions. The coil location and orientation that elicited responses as described in the main text was set as the optimal site and recorded by a frameless, stereotaxic neuronavigation system (Brainsight, Rogue Research Inc., Montreal, Quebec, Canada).

*Transcutaneous Electrical Stimulation*

Electrical stimulation was applied to the skin overlying the brachial plexus at Erb’s point and ulnar nerve just proximal to the ulnar styloid at the wrist. Circular (1 cm diameter) and rectangular (30 cm^2^ diameter) surface electrodes were secured over Erb’s point (cathode) and the region overlying the suprascapular fossa (anode), respectively. Bipolar felt pad electrodes soaked in a saline solution were secured to the wrist with the cathode and anode separated by 2 cm and the cathode positioned opposite the FDI muscle. Foam cushions were placed under the hand and forearm to elevate both from the arm tray and avoid changes in pressure on the stimulating electrode. Electrical current (200 μs pulse duration) was supplied by a constant-current stimulator (DS7R, Digitimer Ltd.). Current was graded higher until the peak-to-peak amplitude of the M-wave saturated and, then, decreased in small increments to established M_MAX_ threshold.

*Triple Stimulation Technique*

The technique involved triggering three stimulation pulses in short succession to produce control and test curves recorded from the paretic FDI muscle. Curves refer to the second waveform elicited from each set of stimuli. The control curve, which serves as a normalization to the test curve, involved pulses of electrical current to the following locations in order: (1) plexus, (2) nerve, and (3) plexus. The test curve involved magnetic and/or electrical pulses to the following locations in order: (1) cortex (magnetic), (2) nerve (electric), and (3) plexus (electric). Volleys elicited by stimulation to the first and second neural element collide in the periphery along the length of the arm. Specific delays are set after each successive stim pulse. Intervals corresponding to the first and second delays while generating the control curve are equal to the plexus M_MAX_ latency minus the nerve M_MAX_ latency. The interval corresponding to the first delay while generating the test curve is equal to the latency of the cortical MEP, rounded down to the nearest millisecond, minus the nerve M_MAX_ latency, rounded up to the nearest millisecond. The second delay is equal to the plexus M_MAX_ latency, rounded down to the nearest millisecond, minus the nerve M-max latency, rounded up to the nearest millisecond.

The first waveform from control and test stimulation is the M_MAX_ from nerve stimulation, and its peak-to-peak amplitude will remain constant since orthodromic volleys directly activate the motor endplate at the neuromuscular junction. The amplitude of the second waveform elicited by the third stimulation pulse to plexus while generating each curve will vary and depends on the extent of collisions between volleys elicited by stimulation to the first and second neural elements. Activation of the plexus is distal to the spinal motor neuron pools and, therefore, brings all axons under the stimulating electrode to threshold. The result in the case of the control curve is that all antidromic volleys elicited by nerve stimulation will cancel after colliding with orthodromic volleys elicited by plexus stimulation, allowing all orthodromic volleys elicited by the third stimulation pulse to propagate to the neuromuscular junction. However, in the case of the test curve wherein activation is *pre-* motor neuronal at the level of the spinal cord, lesions of descending fibers make it unlikely that descending volleys will activate the entire pool of spinal motor neurons. In this case, antidromic volleys elicited by nerve stimulation (i.e., pulse 2) will not be completely cancelled by cortical stimulation (i.e., pulse 1), causing some remainder of antidromic volleys to collide with orthodromic volleys elicited by plexus stimulation (i.e., pulse 3) and limiting propagation of the latter volleys to the neuromuscular junction. In general, the peak-to-peak amplitude of the second waveform in the test curve is dependent on the proportion of spinal motor neurons brought to threshold by cortical stimulation. Ultimately, the greater the proportion of upper motor neurons brought to threshold by cortical stimulation, the greater the number of collisions between descending volleys and antidromic volleys, leading to greater superimposition of the test curve on the control curve. A higher test–control curve ratio, therefore, can be taken to approximate how much of the spinal motor neuron pool is accessible to cortical motor neurons.

After verifying the onset of the rhythmic bursts of muscle activity elicited by single-pulse TMS, the location of Erb’s point was established and electrodes were secured for plexus stimulation. A total of 3-5 compound muscle action potentials (CMAPs) were recorded at M_MAX_ threshold. Next, electrodes were secured over the ulnar nerve and CMAPs were recorded at M_MAX_ threshold. A total of 40 pulses were administered (1 Hz) to the ulnar nerve at M_MAX_ threshold to determine M- and F-wave latencies. The latency from all responses were entered into equations to set timing of triggers for each stimulation device. Prior to obtaining test curves then control curves, the forearm was strapped to the arm tray for safety. Background EMG was streamed in real time to verify that voluntary muscle activity was absent at the time stimulation was administered. A pause was provided between each series of pulses in test and control curves on an as-needed basis. Procedures resumed no sooner than 10 s after the prior pulse series was administered.

*Electrophysiology Data Processing*

All EMG recording were inspected to verify stability of the signal 100 ms prior to onset of stim pulses. Trials were removed from the analysis if peak-to-peak amplitude of the signal in this window was > 2 standard deviations above the mean across all recordings. Using the latency of the aberrant motor response elicited by single-pulse TMS as well as M- and F-wave latencies, peripheral and central conduction times were calculated as follows:

Peripheral = F-wave_Latency_ −M-wave_Latency_ ∗ 0.5

Central = MEP_Latency_ – PCT + M-wave_Latency_

The mean peak-to-peak amplitude of the test curve obtained with the triple stimulation technique was expressed as a percentage of the mean peak-to-peak amplitude of the control curve (Urbin *et al.*, 2021). Mean peak-to-peak amplitudes of M- and F-waves were computed. The F-wave was considered present if peak-to-peak amplitude was >20 μV above background within a 20-ms window at the predetermined latency (Christiansen *et al.*, 2018). F-wave persistence was calculated by taking the percentage of stimulation pulses that elicited a F-wave.

**References**

An KN, Ueba Y, Chao EY, Cooney WP & Linscheid RL (1983). Tendon excursion and moment arm of index finger muscles. *Journal of Biomechanics*, 16(6), 419-425. DOI: 10.1016/0021-9290(83)90074-X.

Buetefisch CM, Revill KP, Haut MW, Kowalski GM, Wischnewski M, Pifer M, Belagaje SR, Nahab F, Cobia DJ, Hu X, Drake D & Hobbs G (2018). Abnormally reduced primary motor cortex output is related to impaired hand function in chronic stroke. *Journal of Neurophysiology*, 120(4),1680-1694. DOI: 10.1152/jn.00715.2017.

Christiansen L, Urbin M, Mitchell GS & Perez MA (2018). Acute intermittent hypoxia enhances corticospinal synaptic plasticity in humans. *Elife*, 7:e34304. DOI: 10.7554/elife.34304.

Johnston JA, Winges SA & Santello M (2009). Neural control of hand muscles during prehension. *Advances in Experimental Medicine and Biology*, 629, 577-596. DOI: 10.1007/978-0-387-77064-2_31.

Kamagata K, Andica C, Uchida W, Takabayashi K, Saito Y, Lukies M, Hagiwara A, Fujita S, Akashi T, Wada A, Hori M, Kamiya K, Zalesky A & Aoki S (2024). Advancements in Diffusion MRI Tractography for Neurosurgery. *Investigative Radiology*, 59, 13-25. DOI: 10.1097/RLI.0000000000001015.

Khan SI, Giesebrecht S, Gandevia SC & Taylor JL (2012). Activity-dependent depression of the recurrent discharge of human motoneurones after maximal voluntary contractions. *Journal of Physiology*, 590, 4957-4969. DOI: 10.1113/jphysiol.2012.235697.

Larsen LH, Jensen T, Christensen MS, Lundbye-Jensen J, Langberg H & Nielsen JB (2016). Changes in corticospinal drive to spinal motoneurones following tablet-based practice of manual dexterity. *Physiological Reports*, 4(2), e12684. DOI: 10.14814/phy2.12684.

Magistris MR, Rösler KM, Truffert A & Myers JP (1998). Transcranial stimulation excites virtually all motor neurons supplying the target muscle: A demonstration and a method improving the study of motor evoked potentials. *Brain*, 121, 437–450. DOI: 10.1093/brain/121.3.437.

Sathian K, Buxbaum J, Cohen G, Krakauer W, Lang E, Corbetta M & Fitzpatrick M (2011).

Neurological principles and rehabilitation of action disorders: Common clinical deficits. *Neurorehabilitation & Neural Repair*, 25, 21-32. DOI: 10.1177/1545968311410941.

Udupa K, Chen R. In: Handbook of Clinical Neurology. Lozano A.M., Hallett M., editors. Elsevier; 2013. Chapter 31 - Central motor conduction time; pp. 375–386. 116.

Urbin MA, Collinger JL & Wittenberg GF (2021). Corticospinal recruitment of spinal motor neurons in human stroke survivors. *Journal of Physiology*, 599(18), 4357-4373. DOI: 10.1113/JP281311.

Usuda N, Sugawara SK, Fukuyama H, Nakazawa K, Amemiya K & Nishimura Y (2022). Quantitative comparison of corticospinal tracts arising from different cortical areas in humans. *Neuroscience Research*, 183, 30-49. DOI: 10.1016/j.neures.2022.06.008.

Vallence AM, Rurak BK, Fujiyama H & Hammond GR (2023). Covariation of the amplitude and latency of motor evoked potentials elicited by transcranial magnetic stimulation in a resting hand muscle. *Experimental Brain Research*, 241(3), 927-936. DOI: 10.1007/s00221-023-06575-z.

Yeh FC, Verstynen TD, Wang Y, Fernández-Miranda JC & Tseng WYI (2013). Deterministic diffusion fiber tracking improved by quantitative anisotropy. *PLoS One*, 8:e80713. DOI: 10.1371/journal.pone.0080713.
